# A fuzzy robust optimization model for dual objective forward and reverse logistics networks considering carbon emissions

**DOI:** 10.1371/journal.pone.0316197

**Published:** 2025-03-11

**Authors:** Yuepeng Shi, Botang Li, Maxim A. Dulebenets, Yui-Yip Lau

**Affiliations:** 1 School of Energy and Intelligent Engineering, Henan University of Animal Husbandry and Economy, Zhengzhou, China; 2 Department of Port and Shipping Management, Guangzhou Maritime University, Guangzhou, China; 3 College of Engineering, Florida A&M University-Florida State University (FAMU-FSU), Tallahassee, Florida, United States of America; 4 Division of Business and Hospitality Management, College of Professional and Continuing Education, The Hong Kong Polytechnic University, Hong Kong, China; Shanghai Jiaotong University: Shanghai Jiao Tong University, CHINA

## Abstract

The inherent unpredictability within the low-carbon integrated supply chain logistics network complicates its management. This paper endeavours to address the challenge of designing a low-carbon logistics network within a context of uncertainty and with consideration of low-carbon policies. It also endeavours to identify locations of facilities and appropriate transportation routes between nodes. Robust optimisation and fuzzy programming techniques are employed to examine the various attributes of the network. In addition, the strategic planning model of a multi-level forward/reverse integration logistics network is examined, with the aims of cost minimisation and emission reduction. Extensive computational simulations substantiate the efficacy of the proposed robust fuzzy programming model. Moreover, analytical results indicate the rationality and applicability of the decisions suggested by the proposed optimisation model and the solution approach. Furthermore, the results indicate that a decision maker can ascertain that the decisions derived from three cases considered have a 50% probability of being the most favourable outcomes.

## 1. Introduction

Early logistics networks were typically unidirectional open-loop structures, such as flows from suppliers to manufacturing plants, from manufacturing plants to distribution centres, and from distribution centres to retailers. The main activities in such networks included procurement of materials or components, production, and distribution of products. Industrial upgrading and increasing attention to ecological issues has led to the proposal of concepts such as green manufacturing, remanufacturing, and the circular economy. Consequently, traditional forward logistics networks are increasingly evolving into integrated forward/reverse logistics networks, i.e., closed-loop structures that integrate forward and reverse logistics activities. Reverse logistics is the opposite process to forward logistics and involves manufacturing plants systematically receiving products and components that have been shipped from the consumer end. These products and components are examined to determine whether they warrant recycling, remanufacturing, or disposal, thereby effectively utilising resources [1]. Therefore, finding a scientific and efficient logistics network has become key to solving the problem.

In reality, many logistics networks are based on existing forward logistics networks. Thus, the layout and design of reverse logistics networks are generated by adding reverse logistics network functional nodes into forward logistics networks or by adding reverse logistics functions onto forward logistics network nodes. As a forward logistics network is inherently complex, the addition of reverse logistics nodes into a forward logistics network affords an integrated forward/reverse logistics network that is even more complex [2]. Therefore, the design and construction of the reverse logistics part of an integrated forward/reverse logistics network attracts much research attention.

Ignoring uncertainty in the design of a reverse logistics network or integrated forward/reverse logistics network affords a static logistics network, such as that associated with large-scale, large-volume centralised production. In such a network, the uncertainty of product quality and quantity of recovered products has a low impact [3]. However, actual manufacturing supply chain logistics networks operate in complex and dynamic environments. Moreover, the complexity and size of these networks are increased by globalisation and the continual refinement of the division of labour. Thus, to deal with the uncertain parameters of the logistics system and the environment in which it operates, scholars have increasingly examined the robustness of supply chain logistics networks. In these networks, changes can occur in random variables in a normal state, such as raw material prices, energy consumption, market demand, labour costs, finished product prices, and exchange rates. These changes have a rather small impact on a supply chain logistics network and can be simulated using standard probability methods. However, some parameters in a supply chain logistics network are difficult to measure, such as whether demand is less than a certain fixed value, and the accuracy of such measurements is unclear.

Countries worldwide are increasingly paying attention to the coordination of economic development with environmental protection. Reverse logistics can effectively reduce environmental pollution and achieve rational and efficient use of energy and resources, thereby reducing waste. Therefore, enterprises implementing low-carbon forward and reverse logistics can reduce pollution and destruction of the environment, save natural resources, shape a positive corporate image, reduce production costs, and enhance their core competitiveness and competitive advantage. Moreover, they can align their operations with the concept of green and circular sustainable economic development [4]. Similarly, designing an integrated forward/reverse logistics network with the dual objectives of minimising logistics costs and carbon emissions has several advantages. Specifically, it can standardise the economic order of the logistics industry to achieve low-cost efficient recycling, promote the efficient use of natural resources, control the emission of carbon dioxide in the reverse logistics process of product recycling, achieve a balance between environmental and economic benefits, and achieve the highest overall benefit.

Accordingly, this paper examines the strategic planning model of a multi-level forward/ reverse integration logistics network, aiming at cost minimisation and emission reduction. To adequately capture uncertainty, randomness, and fuzziness parameters (which are independent of each other), robust optimisation and fuzzy programming methods are directly incorporated within the proposed methodology.

This paper makes the following innovative contributions. (1) It integrates a reverse logistics network into the original, traditional forward logistics network to establish a forward/reverse integrated logistics network. (2) It adds disposal centres and the calculation of carbon emissions into the established forward/reverse integrated logistics network. (3) It pioneers the simultaneous application of robust optimisation methods and fuzzy programming methods to address the uncertainties in a low-carbon forward/reverse integrated logistics network.

The remainder of this paper is organised as follows. Section 2 presents a literature review. Section 3 introduces the problem background and describes the composition of the model. Section 4 describes the numerical experiments, and Section 5 summarises the results.

## 2. Literature review

At present, excessive greenhouse gas emissions caused by high global energy consumption are adversely affecting animals and people’s daily activities [5,6]. With the concept of ‘carbon neutrality’ being part of China’s 14th Five-Year Plan, the logistics industry is facing substantial environmental challenges. In particular, it is required to reduce its carbon emissions by optimising its transportation network [7–9]. Integrated forward/reverse logistics can indirectly reduce the amount of environmental pollution through the reuse of resources. However, emissions of greenhouse gases resulting from energy use in the transportation, recycling, and disposal phases of products have commonly been overlooked in previous studies [10,11]. Therefore, scholars from various countries have begun to conduct preliminary research on the abovementioned issues.

### 2.1. Forward/reverse logistics

Many researchers have examined various practical issues to deepen the understanding of integrated forward/reverse logistics networks. For instance, Prajapati et al. [12] developed a sustainable framework for the transition of the e-commerce industry towards a circular economy in a multi-echelon, multi-product closed-loop supply chain. Their aim was to reduce the total cost associated with the forward and reverse flow of goods in the closed-loop supply chain while maximising revenue, with a focus on sustainability. Zhou et al. [13] designed a multi-echelon reverse logistics network by considering the economic and environmental costs in the recycling process of express packaging waste. This network addressed the capacity-location-routing problem in reverse logistics networks combined with customer incentive mechanisms. Yu et al. [14] proposed an integrated forward/reverse logistics network with consideration of a heterogeneous fleet with different loading capacities and transportation costs. The aim of considering different loading capacities was to deliver the demands of suppliers to customers, while the aim of considering different transportation costs was to return unsold products from customers to suppliers. Yu and Sun [15] presented an integrated and digital architecture for the design of uncertain reverse logistics networks. They also established a fuzzy optimisation model to identify potential network configurations under different demand satisfaction and capacity constraints. Li and Alumur [16] explored the effective management of water resources in the hydraulic fracturing process of natural gas extraction. In particular, they adopted a multi-period reverse logistics network design perspective and realistic modelling methods to determine the optimal locations and capacities of reservoirs, treatment facilities, and disposal sites. These efforts reduced fixed and operational costs, and decreased freshwater usage by 12.8%. Ma et al. [17] proposed a three-level interactive decision-making framework with consideration of product design. Specifically, they established an integrated crowdsourced contracting reverse logistics platform, proposed a three-level mixed 0-1 planning model, and using Stackelberg game theory to handle the complex decision-making process.

However, there is a lack of general research on integrated forward/reverse logistics networks in previous studies.

### 2.2. Carbon emissions

Talaei et al. [18] built a multi-objective low-carbon closed-loop green logistics network fuzzy-planning model for carbon emission reduction. In addition, a few scholars have investigated the interconnection between logistics-related carbon emissions and their influencing factors, such as the mass of transport vehicles and the distances these vehicle travel. For instance, Elhedhli and Merrick [19] developed a model for facility location and distribution to examine the link between carbon emissions and the weight of transportation vehicles. Accorsi et al. [20] created a facility-siting analytical tool for assessing the interplay between carbon emissions and overall expenditure (production and logistics expenses) due to various factors. These factors included product transportation distance and the agro-ecosystem in a food distribution network. He et al. [21] optimised a multi-level reverse logistics network for waste batteries from a circular economy perspective by introducing an expected weighting factor to balance the dual objectives of cost minimisation and carbon emission reduction. They also emphasised the importance of selecting different technology types for facilities. Chen et al. [22] integrated the offline and online trading systems for second-hand mobile phones, with consideration of multi-period, multi-product, and multi-tier characteristics; price-sensitive demand; incentives; and quality. They also proposed a novel multi-objective optimisation framework aimed at maximising profits, carbon emission reductions, and circularity in the recycling and processing process.

In the context of a logistics network, the processes of establishing or decommissioning facilities are both financially burdensome and temporally extensive. As such, the determination of locations for facilities is a strategic decision, and the locations of nodes and the paths between them affect costs and carbon emissions [23,24].

### 2.3. Fuzzy programming

The inherent unpredictability of an eco-friendly comprehensive forward and reverse distribution network means that its management is challenging, but there is limited literature on this topic. Talaei et al. [18] accounted for both the overall expenses and carbon footprint within such a network, in addition to incorporating uncertainties. In addition, they formulated a multi-objective fuzzy programming framework for a cohesive forward/reverse logistics framework. They validated this framework and analysed its feasibility within the context of the photocopier manufacturing sector. Pishvaee et al. [25] formulated a dual-objective fuzzy programming framework aimed at carbon emission reduction and addressing varying capacity constraints within logistics networks. Ghanbarzadeh-Shams et al. [26] introduced a novel fuzzy multi-objective programming model with chance constraints that integrated flexibility and possibility. This model considered the carpet industry’s susceptibility to high uncertainty affecting collection rates, the availability of recyclable items, and reverse channel capacities. Thus, the model addressed the multi-product, multi-site, and multi-period production planning problem under uncertainty in integrated reverse logistics. Tosarkani et al. [27], in the context of recycling plastic beverage containers, considered economic, environmental, and social objectives and established a reverse logistics network model. They also employed a scenario-based possibility approach to handle parameter uncertainty and developed a data-driven fuzzy optimisation framework. Raad and Rajendran [28] emphasised that effective network design is key to success. They also noted that decisions regarding location and capacity must consider objective data and expert opinions to avoid costly long-term consequences. Furthermore, they combined fuzzy multi-criteria decision-making methods with fuzzy stochastic chance-constrained optimisation to form an integrated multi-objective model, which they solved using the epsilon-constraint method.

### 2.4. Robust optimisation

Some scholars have used robust optimisation methods [29] to solve network uncertainty problems. For example, Bertsimas et al. [30,31] proposed new robust optimisation methods based on different sets of uncertain parameters and obtained linear robust pairwise equations through formula transformation. They then applied these equations to logistics network management. Hatefi and Jolai [32] simultaneously considered uncertain parameters and facility interruptions to reduce costs. They also proposed a comprehensive forward/reverse logistics network model that employed integer linear programming and aimed to reduce interruption risk by enhancing robustness criteria. Wang et al. [33] generated a logistics network encompassing both remanufacturing and the integration of forward and reverse flow models to reduce carbon trading revenue and logistics costs under tradable carbon emission rights. They also used robust optimisation methods to deal with uncertainty in the network. However, the abovementioned studies have defined only some model parameters as random or fuzzy, while real logistics networks are complex and involve multiple types of parameters. As such, the uncertainty of parameters in network models should be diverse and defined as random and/or fuzzy. Therefore, multiple types of uncertainty should be the focus of research on logistics networks [34].

In summary, the optimisation of low-carbon forward/reverse integrated logistics networks under uncertain environments is a rather new research topic. As shown in S1. although many researchers have examined this topic, few studies have simultaneously considered carbon emissions, fuzziness, and robustness in general forward/reverse integrated logistics networks. Therefore, this paper optimises a forward/reverse integrated logistics network from the perspective of uncertainty planning and accounts for aspects such as carbon emissions, fuzziness, and robustness.

## 3. Problem description and uncertainty modelling

### 3.1. Problem definition

**Fig 1:**
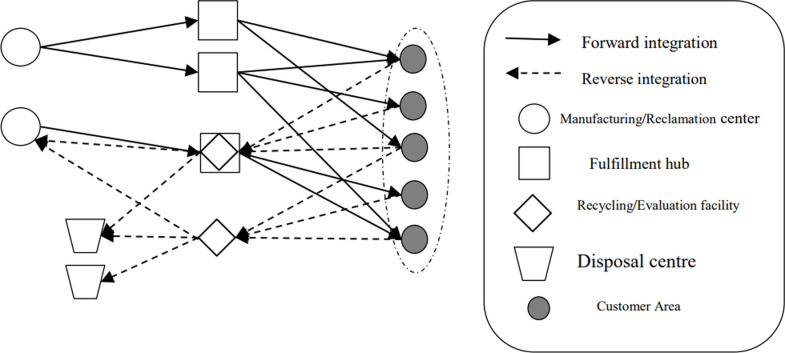
Logistics network structure of forward and reverse integration.

As depicted in Fig 1, the flow of new products begins at the manufacturing/recycling nexus and advances towards the distribution facility on the path to reaching the consumer market. Concurrently, items returned by customers are routed to the recovery/inspection hub for evaluation. There, products deemed recyclable are redirected to the production/recovery facility, while those deemed unrecyclable are sent to a disposal site. The integration of hybrid facilities – where distribution and recovery/inspection functions coexist – presents cost-efficiency benefits over separate operations. These savings are quantified within the goal function within the supply chain network’s optimisation framework, as illustrated in the figure.

Within the logistics network, carbon emissions primarily originate from two distinct sources: (1) the internal energy use of facilities, which encompasses electricity, fuel, and heat use (this paper focuses on electricity, as the principal form of energy consumed); and (2) the transportation phase between facilities, which is influenced by variables such as vehicle mass, travel distance, and road conditions, including gradients and traffic congestion. For simplicity, the paper confines its analysis to the influence of vehicle mass and travel distance. Furthermore, this paper uses symmetric intervals or trapezoidal fuzzy numbers to delineate the fluctuation in uncertain parameters related to network attributes, as elaborated in subsequent sections.

### 3.2. Model assumptions and symbols

For the convenience of modelling, the following conditions are assumed: (1) there is a single-cycle single product; (2) the network’s facilities can be categorised into three tiers of capability, and their fixed operational costs and associated carbon emissions increase in tandem with the level of a tier; (3) the same product generates the same carbon emissions during the operation of the same facility; and (4) customers are willing to use remanufactured products.

Defining symbols:

1. Sets: *I* contains potential locations for production and recovery operations, i∈I *J* contains potential locations for distribution hubs, j∈J *K* contains established locations within the customer region, k∈K Set *L* contains potential locations for recycling and inspection centres, l∈L *M* contains potential locations for disposal centres, m∈M *N* contains the facility’s capacity levels, n∈N and *E* contains potential integration points between recycling/inspection facilities and distribution nodes, e∈E,E⊂J,E⊂L
2. Parameters: ${\tilde{d}}_{k}$ is the fuzzy demand of customer area *k*; ${\tilde{r}}_{k}$ is the fuzzy recovery rate of customer area *k*, a constrained symmetric stochastic variable; $\tilde{s}$ is the average processing rate, a confined symmetric stochastic quantity; $f_{i}^{n}$ is the fixed operational expense associated with production/recovery centre *i* at capacity level *n*; $o_{j}^{n}$ is the inherent cost of the distribution center *j* under level *n*; $h_{l}^{n}$ is the constant expense associated with the recovery/inspection centre *l* at capacity level *n*; $a_{m}^{n}$ is the predetermined expense for waste disposal centre *m* at capacity level *n*; $f_{e}^{nn^{\prime }}$ are the fixed cost savings of the joint point *e* of the distribution centre at capacity level *n* and recovery/inspection center at capacity level *n*^′^; $\tilde{cx},\tilde{cu},\tilde{cq},\tilde{cp}$ are respectively fuzzy unit transportation costs between facilities; ${\tilde{caw}}_{i}^{n}$ is the fuzzy forward capability of production/recovery centre *i* at capacity level *n*; ${\tilde{cay}}_{j}^{n}$ is the fuzzy capability of distribution centre *j* at capacity level *n*; ${\tilde{caz}}_{l}^{n}$ is the fuzzy capability of recovery/inspection centre *l* at capacity level *n*; ${\tilde{cav}}_{m}^{n}$ is the fuzzy capability of disposal centre *m* at capacity level *n*; ${\tilde{car}}_{i}^{n}$ is the reverse capability of production/recovery centre *i* at capacity level *n*; *β* is a substantially large value; $\tilde{\gamma }$ is the fuzzy CO_2_ emission factor, g/km⋅car; $t_{ij}^{1},t_{jk}^{2},t_{kl}^{3},t_{lm}^{4},t_{li}^{5}$ are the distances between facilities, respectively, km; vc is the carrying capacity of a vehicle; $\theta_{i}^{n},\theta_{j}^{n},\theta_{l}^{n},\theta_{m}^{n}$ are the fixed CO_2_ emission factors of initiating a manufacturing/recovery facility *i*, distribution center *j*, recycling/inspection center *l* and disposal center *m*, respectively, g/centre; and ${\tilde{v}}_{1},{\tilde{v}}_{2},{\tilde{v}}_{3},{\tilde{v}}_{4}$ are the fuzzy CO_2_ emission coefficients of unit products processed within the establishment of manufacturing/recovery facilities, fulfillment hubs, recycling/evaluation centres, and waste disposal facilities, g/ piece.
3. Decision variables: $X_{ij},U_{jk},Q_{kl},T_{lm},P_{li}$ are transport volumes between facilities; $W_{i}^{n}$ is a 0-1 variable that takes a value of 1 if the manufacturing and recycling facility *i* with capacity tier *n* is selected, and 0 otherwise; $Y_{j}^{n}$ is a 0-1 contingent factor that takes a value of 1 if distribution centre *j* with a capacity tier *n* is selected, and 0 otherwise; $Z_{l}^{n}$ is a 0-1 variable that takes a value of 1 if the recovery/inspection centre *l* with the ability level *n* is selected, and 0 otherwise; $V_{m}^{n}$ is a 0-1 variable that takes a value of 1 if a disposal center *m* with ability level *n* is selected, and 0 otherwise.

### 3.3. Objective functions and constraints of the optimisation model

1. The first objective function aggregates the fixed establishment expenses and deducts the incurred savings by integrating facilities and transportation costs. Therefore, Objective 1 (fixed opening and logistics cost minimisation) can be defined as follows:

$$\begin{array}{c} \text{Min }w_{1}=\sum_{i\in I}\sum_{n\in N}f_{i}^{n}W_{i}^{n}+\sum_{j\in j}\sum_{n\in N}o_{j}^{n}Y_{j}^{n}+\sum_{l\in L}\sum_{n\in N}h_{l}^{n}Z_{l}^{n}+{\sum_{m\in M}\sum_{n\in N}a_{m}^{n}V_{m}^{n}}_{m}^{n}V_{m}^{n}\\ -\sum_{e\in I}\sum_{n^{\prime }\in N}\sum_{n^{\prime }\in N}f_{i}^{nn^{\prime }}Y_{e}^{n}Z_{e}^{n^{\prime }}+c\tilde{x}\sum_{i\in I}\sum_{j\in J}t_{ij}^{1}X_{ij}+c\tilde{u}\sum_{j\in J}\sum_{k\in K}t_{jk}^{2}U_{jk}\\ +c\tilde{q}\sum_{k\in K}\sum_{l\in L}t_{kl}^{3}Q_{kl}+c\tilde{t}\sum_{l\in L}\sum_{m\in M}t_{lm}^{4}T_{lm}+c\tilde{p}\sum_{l\in L}\sum_{i\in I}t_{li}^{5}P_{li} \end{array}$$ (1)

2. According to the problem description, Objective 2 (carbon emission minimisation) can be defined as follows:

$$\begin{array}{c} \text{Min }w_{2}=\left(\sum_{i\in I}\sum_{j\in J}t_{ij}^{1}\left\lceil \frac{X_{ij}}{vc}\right\rceil +\sum_{j\in J}\sum_{k\in K}t_{jk}^{2}\left\lceil \frac{U_{jk}}{vc}\right\rceil +\sum_{k\in K}\sum_{l\in L}t_{kl}^{3}\left\lceil \frac{Q_{kl}}{vc}\right\rceil +\sum_{l\in L}\sum_{m\in M}t_{lm}^{4}\left\lceil \frac{T_{lm}}{vc}\right\rceil +\sum_{l\in L}\sum_{i\in I}t_{li}^{5}\left\lceil \frac{P_{li}}{vc}\right\rceil \right)\\ +\sum_{n}\sum_{i}\theta_{i}^{n}W_{i}^{n}+\sum_{n}\sum_{j}\theta_{j}^{n}Y_{j}^{n}+\sum_{n}\sum_{l}\theta_{i}^{n}Z_{l}^{n}+\sum_{n}\sum_{m}\theta_{m}^{n}V_{m}^{n}\\ +{\tilde{v}}_{1}\left(\sum_{i\in I}\sum_{j\in J}X_{ij}+\sum_{l\in L}\sum_{i\in I}P_{li}\right)+{\tilde{v}}_{2}\sum_{j\in J}\sum_{k\in K}U_{jk}+{\tilde{v}}_{3}\sum_{k\in K}\sum_{l\in L}Q_{kl}+{\tilde{v}}_{4}\sum_{l\in L}\sum_{m\in M}T_{lm} \end{array}$$ (2)

According to the assumptions adopted, the decision variables must meet the following constraints:


$$\underset{j\in J}{\sum }U_{jk}\geq \tilde{d_{k}},\forall k\in K$$ (3)

$$\underset{l\in L}{\sum }Q_{kl}\geq \tilde{r_{k}}\underset{j\in J}{\sum }U_{jk},\forall k\in K$$ (4)

$$\underset{i\in I}{\sum }X_{ij}\geq \underset{k\in K}{\sum }U_{jk},\forall j\in J$$ (5)

$$\underset{m\in M}{\sum }T_{lm}\geq \tilde{s}\underset{k\in K}{\sum }Q_{kl},\forall l\in L$$ (6)

$$\underset{i\in I}{\sum }P_{li}\geq \left(1-\tilde{s}\right)\underset{k\in K}{\sum }Q_{kl},\forall l\in L$$ (7)

$$\underset{j\in J}{\sum }X_{ij}\leq \underset{n\in N}{\sum }W_{i}^{n}c\tilde{a}w_{i}^{n},\forall i\in I$$ (8)

$$\underset{i\in I}{\sum }X_{ij}\leq \underset{n\in N}{\sum }Y_{j}^{n}c\tilde{a}y_{j}^{n},\forall j\in J$$ (9)

$$\underset{k\in K}{\sum }U_{jk}\leq \underset{n\in N}{\sum }Y_{j}^{n}c\tilde{a}y_{j}^{n},\forall j\in J$$ (10)

$$\underset{k\in K}{\sum }Q_{kl}\leq \underset{n\in N}{\sum }Z_{l}^{n}c\tilde{a}z_{l}^{n},\forall l\in L$$ (11)

$$\underset{l\in L}{\sum }T_{lm}\leq \underset{n\in N}{\sum }V_{m}^{n}c\tilde{a}v_{m}^{n},\forall m\in M$$ (12)

$$\underset{l\in L}{\sum }P_{li}\leq \underset{n\in N}{\sum }W_{i}^{n}c\tilde{a}r_{i}^{n},\forall i\in I$$ (13)

$$\underset{m\in M}{\sum }T_{lm}+\underset{i\in I}{\sum }P_{li}\leq \underset{n\in N}{\sum }Z_{l}^{n}c\tilde{a}z_{l}^{n},\forall l\in L$$ (14)

$$\underset{l\in L}{\sum }P_{li}\leq \beta \underset{j\in J}{\sum }X_{ij},\forall i\in I$$ (15)

$$\underset{n\in N}{\sum }W_{i}^{n}\leq 1,\forall i\in I$$ (16)

$$\underset{n\in N}{\sum }Y_{j}^{n}\leq 1,\forall j\in J$$ (17)

$$\underset{n\in N}{\sum }Z_{l}^{n}\leq 1,\forall l\in L$$ (18)

$$\underset{n\in N}{\sum }V_{m}^{n}\leq 1,\forall m\in M$$ (19)

$$W_{i}^{n},Y_{j}^{n},Z_{l}^{n},V_{m}^{n}\in \left\{0,1\right\},\forall i\in I,\forall j\in J,l\in L,m\in M,n\in N$$ (20)

$$X_{ij},U_{jk},Q_{kl},P_{li},T_{lm}\geq 0,\forall i\in I,j\in J,k\in K,l\in L,m\in M$$ (21)

Constraints (3) and (4) ensure that customer demand is met and that a certain proportion of the products is recycled, respectively. Constraints (5) to (7) indicate the flow balance. Constraints (8) to (15) indicate the facility capacity. Constraints (16) to (19) indicate that a certain capacity level should be selected for each open facility. Constraint (20) indicates the integrality of binary variables, and Constraint (21) indicates the integrality of non-negative variables.

As the nonlinearity is caused by $\left\lceil \frac{X_{ij}}{vc}\right\rceil$, a supplementary variable $X_{ij}^{1}$ is added into the model, as follows:


$$X_{ij}^{1}\geq \frac{X_{ij}}{vc},\forall i,j$$ (22)

$$X_{ij}^{1}\leq \frac{X_{ij}}{vc}+\varepsilon ,\forall i,j$$ (23)

$$\text{ε}\to 1^{-},\forall i,j,k,l,m,n,e$$ (24)

$$X_{ij}^{1}\in N\cup \left\{0\right\},\forall i,j$$ (25)

The treatment of $\left\lceil \frac{U_{jk}}{vc}\right\rceil$, $\left\lceil \frac{Q_{kl}}{vc}\right\rceil$, $\left\lceil \frac{T_{lm}}{vc}\right\rceil$ and $\left\lceil \frac{P_{li}}{vc}\right\rceil$ is performed in the same manner, and supplementary variables $U_{jk}^{1}$, $Q_{kl}^{1}$, $T_{lm}^{1}$ and $P_{li}^{1}$ are added into the model. Subsequently, Objective 2 is redefined as follows:


$$\begin{array}{c} \text{Min }w_{2}^{*}=\gamma \left(\sum_{i\in I}\sum_{j\in J}t_{ij}^{1}X_{ij}^{1}+\sum_{j\in J}\sum_{k\in K}t_{jk}^{2}U_{jk}^{1}+\sum_{k\in K}\sum_{l\in L}t_{kl}^{3}Q_{kl}^{1}+\sum_{l\in L}\sum_{m\in M}t_{lm}^{4}T_{lm}^{1}+\sum_{l\in L}\sum_{i\in I}t_{li}^{5}P_{li}^{1}\right)\\ +\sum_{n}\sum_{i}\theta_{i}^{n}W_{i}^{n}+\sum_{n}\sum_{j}\theta_{j}^{n}Y_{j}^{n}+\sum_{n}\sum_{l}\theta_{i}^{n}Z_{l}^{n}+\sum_{n}\sum_{m}\theta_{m}^{n}V_{m}^{n}\\ +{\tilde{v}}_{1}\left(\sum_{i\in I}\sum_{j\in J}X_{ij}+\sum_{l\in L}\sum_{i\in I}P_{li}\right)+{\tilde{v}}_{2}\sum_{j\in J}\sum_{k\in K}U_{jk}+{\tilde{v}}_{3}\sum_{k\in K}\sum_{l\in L}Q_{kl}+{\tilde{v}}_{4}\sum_{l\in L}\sum_{m\in M}T_{lm} \end{array}$$ (26)

Furthermore, the term $\sum_{e\in E}\sum_{n^{\prime }\in N}\sum_{n\in N}f_{e}^{nnn^{'}}Z_{e}^{n^{\prime }}Y_{e}^{n}$ in Objective 1 is non-linear. To circumvent the intricacies of mixed-integer non-linear programming, a new variable $Q_{e}^{nn^{\prime }}$ is introduced, and the objective function is linearised as follows:


$$\begin{array}{l} Q_{e}^{nn^{\prime }}=Y_{e}^{n}\times Z_{e}^{n^{\prime }}\\ Q_{e}^{nn^{\prime }}\in \left\{0,1\right\},\forall e\in E,\forall n\in N,\forall n^{\prime }\in N \end{array}$$ (27)

$$\begin{array}{c} \text{Min }w_{1}^{*}=\sum_{i\in I}\sum_{n\in N}f_{i}^{n}W_{i}^{n}+\sum_{j\in J}\sum_{n\in N}o_{j}^{n}Y_{j}^{n}+\sum_{l\in L}\sum_{n\in N}h_{l}^{n}Z_{l}^{n}\\ +\sum_{m\in M}\sum_{n\in N}a_{m}^{n}V_{m}^{n}-\sum_{e\in E}\sum_{n^{\prime }\in E}\sum_{n\in N}f_{e}^{nn^{\prime }}Q_{e}^{nn^{\prime }}\\ +cx\sum_{i\in I}\sum_{j\in J}t_{ij}^{1}X_{ij}+cu\sum_{j\in J}\sum_{k\in K}t_{jk}^{2}U_{jk}+cq\sum_{k\in K}\sum_{l\in L}t_{kl}^{3}Q_{kl}\\ +ct\sum_{l\in L}\sum_{m\in M}t_{lm}^{4}T_{lm}+cp\sum_{l\in L}\sum_{i\in I}t_{li}^{5}P_{li} \end{array}$$ (28)

The following constraint set is added into the updated model formulation:


$$2Q_{e}^{nn^{\prime }}\leq Y_{e}^{n}+Z_{e}^{n^{\prime }},\forall e\in E,n\in N,n^{\prime }\in N$$ (29)

Formula (29) indicates that when $Y_{e}^{n}$ and/or $Z_{e}^{n^{\prime }}$ are both equal to 0 or at least one of them is 0, then the value of $Q_{e}^{nn^{\prime }}$ is 0. When both terms are equal to 1, the value of $Q_{e}^{nn^{\prime }}$ is 1.

### 3.4. Defuzzified model formulation

Objective functions (1) and (2) and Constraints (3,4,6)–(14) of the model parameters outlined in the preceding section incorporate elements of fuzziness. Thus, to facilitate calculation, these objective functions and constraints are converted into their equivalent deterministic forms. According to the literature [18], the necessity transformation can be employed within the framework of probabilistically constrained fuzzy programming to reformulate a target function and particular limitations within an optimisation framework, particularly when dealing with parameters characterised by uncertainty. The optimality of the necessity transformation can be defined as the necessity optimal solution based on randomness theory. In simple terms, the necessity optimal solution is optimal only if all possible solutions are solvable, which is meaningful for meeting chance constraints. Suppose that the linear optimisation model is as follows:


$$\begin{array}{l} \text{Min }Z=fy+cx\\ s.t.Ax\geq d,Sx\leq Ny,Bx=0\\ y\in \left\{0,1\right\},x\geq 0 \end{array}$$

In addition, suppose that the vector *f* is the deterministic parameter, and that the vector sum and coefficient matrix is the indeterminate parameter of the problem. In this case, the objective function is reformulated deterministically by employing the ‘expected value’ approach, while the constraints are translated into their deterministic fuzzy chance equivalents by application of the necessity transformation. Thus, the aforementioned model is reformulated in the following manner:


$$\begin{array}{l} \text{Min E}\left[Z\right]=E\left[f\right]y+E\left[\tilde{c}\right]x\\ s.t.Nec\left\{Ax\geq d\right\}\geq \alpha_{m},\forall m\in M\\ Nec\left\{Sx\leq Ny\right\}\geq \alpha_{m},\forall m\in M\\ Bx=0,y\in \left\{0,1\right\},x\geq 0 \end{array}$$

Herein, the minimum degree of constraint satisfaction is defined, with its value bounded by the interval [0, 1]. Parameters exhibiting uncertainty are characterised by trapezoidal imprecise numerical values, and the corresponding deterministic model is delineated as follows:


$$\begin{array}{l} \text{Min E}\left[Z\right]=fy+\left(\frac{c_{\left(1\right)}+c_{\left(2\right)}+c_{\left(3\right)}+c_{\left(4\right)}}{4}\right)x\\ s.t.Ax\geq \left(1-\alpha_{m}\right)d_{\left(3\right)}+\alpha_{m}d_{\left(4\right)},\forall m\in M\\ Sx\leq \left[\left(1-\alpha_{m}\right)N_{\left(2\right)}+\alpha_{m}N_{\left(1\right)}\right]y,\forall m\in M\\ Bx=0,y\in \left\{0,1\right\},x\geq 0 \end{array}$$

Therefore, the equations in the model containing trapezoidal fuzzy parameters can be converted into their corresponding deterministic equivalents, as follows:


$$\begin{array}{c} \text{Min }w_{1}=\sum_{i\in I}\sum_{n\in N}f_{i}^{n}W_{i}^{n}+\sum_{j\in J}\sum_{n\in N}o_{j}^{n}Y_{j}^{n}+\sum_{l\in L}\sum_{n\in N}a_{m}^{n}V_{m}^{n}\\ -\sum_{e\in E}\sum_{n^{\prime }\in N}\sum_{n\in N}f_{e}^{nm^{\prime }}Q_{e}^{nm^{\prime }}+\left(\frac{cx_{\left(1\right)}+cx_{\left(2\right)}+cx_{\left(3\right)}+cx_{\left(4\right)}}{4}\right)\sum_{i\in I}\sum_{j\in J}t_{ij}^{1}X_{ij}\\ +\frac{cu_{\left(1\right)}+cu_{\left(2\right)}+cu_{\left(3\right)}+cu_{\left(4\right)}}{4}\sum_{j\in J}\sum_{k\in K}t_{jk}^{2}U_{jk}\\ +\frac{cq_{\left(1\right)}+cq_{\left(2\right)}+cq_{\left(3\right)}+cq_{\left(4\right)}}{4}\sum_{k\in K}\sum_{l\in L}t_{kl}^{3}Q_{kl}\\ +\frac{ct_{\left(1\right)}+ct_{\left(2\right)}+ct_{\left(3\right)}+ct_{\left(4\right)}}{4}\sum_{l\in L}\sum_{m\in M}t_{lm}^{4}T_{lm}\\ +\frac{cp_{\left(1\right)}+cp_{\left(2\right)}+cp_{\left(3\right)}+cp_{\left(4\right)}}{4}\sum_{l\in L}\sum_{i\in I}t_{li}^{5}P_{li} \end{array}$$ (30)

$$\begin{array}{c} \text{Min }w_{2}^{*}=\frac{\gamma_{\left(1\right)}+\gamma_{\left(2\right)}+\gamma_{\left(3\right)}+\gamma_{\left(4\right)}}{4} (\sum_{i\in I}\sum_{j\in J}t_{ij}^{1}X_{ij}^{1}+\sum_{j\in J}\sum_{k\in K}t_{jk}^{2}U_{jk}^{1}\\ \left.+\sum_{k\in K}\sum_{l\in L}t_{kl}^{3}Q_{kl}^{1}+\sum_{l\in L}\sum_{m\in M}t_{lm}^{4}T_{lm}^{1}+\sum_{l\in L}\sum_{i\in I}t_{li}^{5}P_{li}^{1}\right)\\ +\sum_{n}\sum_{i}\theta_{i}^{n}W_{i}^{n}+\sum_{n}\sum_{j}\theta_{j}^{n}Y_{j}^{n}+\sum_{n}\sum_{l}\theta_{i}^{n}Z_{l}^{n}\\ +\sum_{n}\sum_{m}\theta_{m}^{n}V_{m}^{n}\frac{\nu_{1_{\left(1\right)}}+\nu_{1_{\left(2\right)}}+\nu_{1_{\left(3\right)}}+\nu_{1_{\left(4\right)}}}{4}\left(\sum_{i\in I}\sum_{j\in J}X_{ij}+\sum_{l\in L}\sum_{i\in I}P_{li}\right)\\ +\frac{\nu_{2_{\left(1\right)}}+\nu_{2_{\left(2\right)}}+\nu_{2_{\left(3\right)}}+\nu_{2_{\left(4\right)}}}{4}\sum_{j\in J}\sum_{k\in K}U_{jk}\\ +\frac{\nu_{3_{\left(1\right)}}+\nu_{3_{\left(2\right)}}+\nu_{3_{\left(3\right)}}+\nu_{3_{\left(4\right)}}}{4}\sum_{k\in K}\sum_{l\in L}Q_{kl}\\ +\frac{\nu_{4_{\left(1\right)}}+\nu_{4_{\left(2\right)}}+\nu_{4_{\left(3\right)}}+\nu_{4_{\left(4\right)}}}{4}\sum_{l\in L}\sum_{m\in M}T_{lm} \end{array}$$ (31)

$$\underset{j\in J}{\sum }U_{jk}\geq \left(1-\alpha_{1}\right)d_{k\left(3\right)}+\alpha_{1}d_{k\left(4\right)},\forall k\in K$$ (32)

$$\underset{j\in J}{\sum }X_{ij}\leq \underset{n\in N}{\sum }W_{i}^{n}\left[\left(1-\alpha_{2}\right)caw_{i\left(2\right)}^{n}+\alpha_{2}caw_{i\left(1\right)}^{n}\right],\forall i\in I$$ (33)

Xj∈Jij≤n∈NYjn[(1−α3)cayj(2)n+α3cayj(1)n],∀j∈J (34)

Uk∈Kjk≤n∈NYjn[(1−α3)cayj(2)n+α3cayj(1)n],∀j∈J (35)

$$\underset{k\in K}{\sum }Q_{kl}\leq \underset{n\in N}{\sum }Z_{l}^{n}\left[\left(1-\alpha_{4}\right)caz_{l\left(2\right)}^{n}+\alpha_{4}caz_{l\left(1\right)}^{n}\right],\forall l\in L$$ (36)

$$\underset{l\in L}{\sum }T_{lm}\leq \underset{n\in N}{\sum }V_{m}^{n}\left[\left(1-\alpha_{5}\right)cav_{m\left(2\right)}^{n}+\alpha_{5}cav_{m\left(1\right)}^{n}\right],\forall m\in M$$ (37)

$$\underset{l\in L}{\sum }P_{li}\leq \underset{n\in N}{\sum }W_{i}^{n}\left[\left(1-\alpha_{6}\right)car_{i\left(2\right)}^{n}+\alpha_{6}car_{i\left(1\right)}^{n}\right],\forall i\in I$$ (38)

$$\underset{m\in M}{\sum }T_{lm}+\underset{l\in L}{\sum }P_{li}\leq \underset{n\in N}{\sum }Y_{l}^{n}\left[\left(1-\alpha_{4}\right)caz_{l\left(2\right)}^{n}+\alpha_{4}caz_{l\left(1\right)}^{n}\right],\forall l\in L$$ (39)

$$0.5\leq \alpha_{b}\leq 1,\forall b=1,2,\ldots ,6$$ (40)

Equations (33)–(39) are inherently non-linear and thus must be linearised to mitigate the escalation of computational complexity within the optimisation model. Therefore, Equation (33) becomes


$$\underset{j\in J}{\sum }X_{ij}\leq \underset{n\in N}{\sum }\left[\left(W_{i}^{n}-\rho_{i}^{\left(1\right)n}\right)caw_{i\left(2\right)}^{n}+\rho_{i}^{\left(1\right)n}caw_{i\left(1\right)}^{n}\right],\forall i\in I$$

$$\rho_{i}^{\left(1\right)n}\leq \beta \cdot W_{i}^{n},\forall n\in N,i\in I$$

$$\rho_{i}^{\left(1\right)n}\geq \beta \cdot \left(W_{i}^{n}-1\right)+\alpha_{2},\forall n\in N,i\in I$$

$$\rho_{i}^{\left(1\right)n}\leq \alpha_{2},\forall n\in N,i\in I$$ (41)

Similarly, let $\rho_{i}^{\left(2\right)n}=Y_{j}^{n}\alpha_{3}$, $\rho_{l}^{\left(3\right)n}=Z_{l}^{n}\alpha_{4}$, $\rho_{m}^{\left(4\right)n}=V_{m}^{n}\alpha_{5}$, $\rho_{i}^{\left(5\right)n}=W_{i}^{n}\alpha_{6}$. Consequently, the linear variations of Equations (34)–(39) can be derived based on Equation (41). The robust transformation can be applied for Constraints (4,6), and (7), based on concepts reported in the literature [29–33], as described below.

In Constraint (4), the recovery rate $r_{k}$is subject to uncertainty and is treated as a constrained symmetric stochastic variable $\tilde{r_{k}}$ with a designated value $\left[r_{k}-\hat{r_{k}},r_{k}+\hat{r_{k}}\right]$, where, $r_{k}$ is the expected value, and $\hat{r_{k}}$ is the upper limit of deviation from this expected value. For each constraint *k*, this paper introduces a new parameter ${\text{Γ}}_{k}^{1}$ value at $\left[0,\left|J_{k}\right|\right]$, where $J_{k}$ represents the quantity of uncertainties in the recovery rate in the first constraint. ${\text{Γ}}_{k}^{1}$ represents the estimated amount of recovery uncertainty used to adjust the conservatism of the model, where recovery uncertainty is related to the decision maker’s estimation of the customer’s willingness and the quality of the recovered product. In practice, all recovery rates cannot be uncertain. Thus for each customer, the recovery rate ${\text{Γ}}_{k}^{1}$ of the product from the various distribution centres varies within the interval $\left[r_{k}-\hat{r_{k}},r_{k}+\hat{r_{k}}\right]$ and within $\left[r_{k}-\left({\text{Γ}}_{k}^{1}-{\text{Γ}}_{k}^{1}\right)\hat{r_{k}},r_{k}+\left({\text{Γ}}_{k}^{1}+{\text{Γ}}_{k}^{1}\right)\hat{r_{k}}\right]$ and does not specify which change. Therefore, the robust correspondence for Constraint (4) can be written as follows:


$$\underset{l\in L}{\sum }Q_{kl}\geq r_{k}\underset{j\in J}{\sum }U_{jk}+\begin{array}{c} max\\ \left\{S_{k}\cup \left\{t_{k}\right\}|S_{k}\subseteq J_{k},\left|S_{k}\right|={\text{Γ}}_{k}^{1},t_{k}\in J_{k}\backslash S_{k}\right\} \end{array}\left\{\underset{j\in S_{k}}{\sum }\hat{r_{k}}y_{jk}^{1}+\left({\text{Γ}}_{k}^{1}-{\text{Γ}}_{k}^{1}\right)\hat{r_{k}}y_{jt_{k}}^{1}\right\},\forall k\in K$$

$$-y_{jk}^{1}\leq U_{jk}\leq y_{jk}^{1},\forall j\in J,k\in K.$$

$$y_{jk}^{1}\geq 0,\forall j\in J,k\in K$$ (42)

where $y_{jk}^{1}$ is the variable added to the restricted value $U_{jk}$.

Given $U_{jk}^{*}$, if ream


$$\beta_{k}\left(U_{jk}^{*},{\text{Γ}}_{k}^{1}\right)=\begin{array}{c} max\\ \left\{S_{k}\cup \left\{t_{k}\right\}|S_{k}\subseteq J_{k},\left|S_{k}\right|={\text{Γ}}_{k}^{1},t_{k}\in J_{k}\backslash S_{k}\right\} \end{array}\left\{\underset{j\in S_{k}}{\sum }\hat{r_{k}}y_{jk}^{1}+\left({\text{Γ}}_{k}^{1}-{\text{Γ}}_{k}^{1}\right)\hat{r_{k}}y_{jt_{k}}^{1}\right\}$$ (43)

then $\beta_{k}\left(U_{jk}^{*},{\text{Γ}}_{k}^{1}\right)$ is equivalent to the objective of the following inquiry:


$$\max\beta_{k}\left(U_{jk}^{*},{\text{Γ}}_{k}^{1}\right)=\underset{j\in J_{k}}{\sum }\hat{r_{k}}\left|U_{jk}^{*}\right|q_{jk}$$

$$s.t.\underset{j\in J_{k}}{\sum }q_{jk}\leq {\text{Γ}}_{k}^{1},\forall k\in K$$

$$0\leq q_{jk}\leq 1,\forall j\in J_{k},k\in K$$ (44)

where $q_{jk}$ is the variable.

Let $\left|J_{k}\right|=\text{NJ}$, soj∈J. Then, according to the duality theory, the robust correspondence of Constraint (4) is transformed into:


$$\underset{l\in L}{\sum }Q_{kl}\geq r_{k}\underset{j\in J}{\sum }U_{jk}+{\text{Γ}}_{k}^{1}z_{k}^{1}+\underset{j\in J}{\sum }p_{jk}^{1},\forall k\in K$$

$$z_{k}^{1}+p_{jk}^{1}\geq \hat{r_{k}}y_{jk}^{1},\forall j\in J,k\in K$$

$$-y_{jk}^{1}\leq U_{jk}\leq y_{jk}^{1},\forall j\in J,k\in K$$

$$y_{jk}^{1},z_{jk}^{1},p_{jk}^{1}\geq 0,\forall j\in J,k\in K$$ (45)

where, $z_{jk}^{1}$ and $p_{jk}^{1}$ are the variables added in accordance with duality theory.

Similarly, consider that *s* in Constraints (6) and (7) is symmetric random variable $\tilde{s}$ with values of $\left[s-\hat{s},s+\hat{s}\right]$. For each constraint, this paper introduces new parameters ${\text{Γ}}_{l}^{2}$ and ${\text{Γ}}_{l}^{3}$ with values of $\left[0,\left|J_{l}\right|\right]$. ${\text{Γ}}_{l}^{2}$ and ${\text{Γ}}_{l}^{3}$ represent the estimated amount of the processing rate uncertainty, which is related to the decision maker’s estimation of the quality of the recovered product and is used to adjust the conservatism of the model.

Following the same methodology, the robust correspondence of Constraint (6) is converted into


$$\underset{m\in M}{\sum }T_{lm}\geq s\underset{k\in K}{\sum }Q_{kl}+{\text{Γ}}_{l}^{2}z_{l}^{2}+\underset{k\in K}{\sum }p_{kl}^{2},\forall l\in L$$

$$z_{l}^{2}+p_{kl}^{2}\geq \;\hat{s}y_{kl}^{2},\forall k\in K,l\in L$$

$$-y_{kl}^{2}\leq Q_{kl}\leq y_{kl}^{2},\forall k\in K,l\in L$$

$$y_{kl}^{2},z_{kl}^{2},p_{kl}^{2}\geq 0,\forall k\in K,l\in L$$ (46)

Similarly, the robust correspondence of Constraint (7) is converted into


$$\underset{i\in I}{\sum }P_{li}+s\underset{k\in K}{\sum }Q_{kl}-{\text{Γ}}_{l}^{3}z_{l}^{3}-\underset{k\in K}{\sum }p_{kl}^{3}\geq \underset{k\in K}{\sum }Q_{kl},\forall l\in L$$

$$z_{l}^{3}+p_{kl}^{3}\geq \hat{s}y_{kl}^{3},\forall k\in K,l\in L$$

$$-y_{kl}^{3}\leq Q_{kl}\leq y_{kl}^{3},\forall k\in K,l\in L$$

$$y_{kl}^{3},z_{kl}^{3},p_{kl}^{3}\geq 0,\forall k\in K,l\in L$$ (47)

In summary, a fuzzy robust programming model is obtained using Equations (30) and (31) as objective functions and using the constraints represented by Equations (5), (15)–(22), (29), (32), (40), (41), (45)–(47).

### 3.5. Transformation of the objective functions

As has been described in previous studies [35,36], there are three principal approaches for addressing multi-objective optimisation dilemmas: the a priori approach, the a posteriori approach, and the interactive approach. In the a priori approach, the decision maker articulates a preference, or weighting, for each objective prior to the optimisation model’s resolution, thereby converting the multi-objective optimisation challenge into a singular, unified objective. In the a posteriori approach, all objective functions are simultaneously optimised to yield an ensemble of efficient solutions (Pareto-efficient solutions). Subsequently, upon the conclusion of the search process, the decision maker identifies and selects the most appropriate solution from this ensemble. In the interactive method, the decision maker’s preferences for each objective are conveyed throughout the participatory decision-making process. The calculation process is interchangeable, and after several iterations, a suitable scheme is selected. In this paper, a compromise solution approach, namely the a posteriori method, is selected for model resolution. Considering that the span of each objective function encompasses the extremes (i.e., the ceiling and floor values), the target function is delineated as follows:


$$\text{Min }w^{*}=\text{δ}\left(\frac{w_{1}^{*}-w_{1}^{*min}}{w_{1}^{*max}-w_{1}^{*min}}\right)+\left(1-\delta \right)\left(\frac{w_{2}^{*}-w_{2}^{*min}}{w_{2}^{*max}-w_{2}^{*min}}\right)$$ (48)

where 0≤δ≤1

The importance of objectives is generally set by decision-makers based on their experience. This paper employs the approach that has been outlined in previous studies [37,38] to ascertain the viability of the dual objectives. Table 1 presents the initial objective function for this approach and its optimal solution.

**Table 1:** Game table.

	*Z*_1_(*X*)	*Z*_2_(*X*)	…	*Z*_k_(*X*)
*X* ^(1)^	*Z*_11_	*Z*_12_	…	*Z*_1k_
*X* ^(2)^	*Z*_21_	*Z*_22_	…	*Z*_2k_
…	…	…	…	…
*X* ^(k)^	*Z*_k1_	*Z*_k1_	…	*Z*_kk_

The minimum and maximum bounds for the optimisation and maximisation problems, respectively, are established by analysing the matrix in Table 2 and applying Equations (49) and (50).

**Table 2:** Summary of the literature review.

Paper	Forward logistics	Reverse logistics	Fuzzy variable	Random variable	Robust optimization	Carbon consideration
Prajapati et al. [12]	Yes	Yes	No	No	No	Yes
Zhou et al. [13]	No	Yes	No	No	No	Yes
Yu et al. [14]	Yes	Yes	No	No	No	No
Yu and Sun [15]	No	Yes	Yes	No	No	No
Li and Alumur [16]	No	Yes	No	Yes	No	No
Ma et al. [17]	No	Yes	No	No	No	No
Elhedhli and Merrick [19]	Yes	No	No	No	No	Yes
Accorsi et al. [20]	Yes	No	No	No	No	Yes
He et al. [21]	No	Yes	No	No	No	Yes
Ghanbarzadeh-Shams et al. [26]	No	Yes	Yes	No	No	No
Tosarkani et al. [27]	No	Yes	Yes	No	No	Yes
Raad and Rajendran [28]	Yes	Yes	Yes	Yes	No	No
Wang et al. [33]	Yes	Yes	No	No	Yes	Yes
This research	Yes	Yes	Yes	No	Yes	Yes


$${\text{Z}}_{\text{k}}^{\text{l}}={\text{Z}}_{\text{kk}}$$

and


$${\text{Z}}_{\text{k}}^{\text{ll}}=\max\left({\text{Z}}_{1\text{k}},{\text{Z}}_{2\text{k}},\ldots ,{\text{Z}}_{\text{kk}}\right)$$ (49)

$$Z_{k}^{ll}=Z_{kk}$$

and


$$Z_{k}^{l}=\min\left(Z_{1k},Z_{2k},\ldots ,Z_{kk}\right)$$ (50)

## 4. Computational experiments

### 4.1. Input data generation

To demonstrate the real-world application of the discussed analytical technique, this paper performs a case analysis of an automobile manufacturer, referred to as Ws company. Ws company relies on the existing engine consumption area of its parent company to recover and repair used engines. Thus, it is necessary to build a network for production/recovery, distribution, recycling/inspection, and disposal. Hence, a reliable strategic plan is needed as a guide. In this case analysis, the facility’s capacity is categorised into three distinct levels, with the cost and carbon emissions measured in yuan and grams (g), respectively. The average processing rate is set to 0.2, while the joint points of the distribution centre and the recycling/inspection centre are J_2_ and L_2_、J_4_ and L_4_、J_5_ and L_5_. The weight *δ* is 0.5, and the capacity of the transportation vehicle is set to five pieces. The other data required for the proposed optimisation model are specified in Tables 3–11.

**Table 3:** Pertinent information of the customer region.

Customer Area	1	2	3	4	5	6	7	8	9	10
Coordinates	(38)	(34,93)	(49,142)	(98,159)	(67,214)	(125,224)	(143,124)	(109,62)	(201,103)	(203,179)
requirements	(50,60, 70,80)	(100,110, 120,130)	(80,90, 100,110)	(50,60, 70,80)	(90,100, 110,120)	(60,70, 80,90)	(80,90, 100,110)	(50,60, 70,80)	(70,80, 90,100)	(60,70, 80,90)
Recovery rates	0.75	0.86	0.83	0.76	0.79	0.87	0.75	0.8	0.75	0.83

**Table 4:** Distribution centre-related data.

Distribution center	1	2	3	4	5
Coordinates	(40,147)	(55,168)	(120,60)	(155,119)	(190,132)
LV 3 fixed cost (ten thousand yuan)	20,25,30	25,30,35	30,35,40	35,40,45	20,25,30
LV 1 capability	(600,650,700,750)	(650,700,750,800)	(700,750,800,850)	(750,800,850,900)	(800,850,900,950)
LV 2 capability	(675,725,775,825)	(725,775,825,875)	(775,825,875,925)	(825,875,925,975)	(875,925,975,1050)
LV 3 capability	(750,800,850,900)	(800,850,900,950)	(850,900,950,1000)	(900,950,1000,1050)	(950,1000,1050,1100)
LV 3 carbon emission	82000,82250,82500	82250,82500, 82750	82500,82750, 83000	82750, 83000,83250	83000,83250,83500

**Table 5:** Recycling/inspection centre-related data.

Recycling/inspection center	1	2	3	4	5
Coordinates	(54,128)	(55,168)	(130,64)	(98,159)	(155,119)
LV 3 fixed cost (ten thousand yuan)	25,30,35	30,35,40	35,40,45	40,45,50	25,30,35
LV 1 capability	(450,500,550,600)	(500,550,600,650)	(550,600,650,700)	(250,300,350,400)	(200,250,300,350)
LV 2 capability	(500,550,600,650)	(550,600,650,700)	(600,650,700,750)	(300,350,400,450)	(250,300,350,400)
LV 3 capability	(550,600,650,700)	(600,650,700,750)	(650,700,750,800)	(350,400,450,500)	(300,350,400,450)
LV 3 carbon emission	820000,822500, 825000	822500,825000,827500	825000,827500,830000	827500,830000,832500	830000,832500,835000

**Table 6:** Production/recovery centre-related data.

Production/recovery centre	1	2	3
Coordinates	(73,151)	(336,224)	(420,138)
LV 3 fixed cost (ten thousand yuan)	45,50,55	50,55,60	55,60,65
LV 1 positive capability	(650,700,750,800)	(700,750,800,850)	(750,800,850,900)
LV 2 positive capability	(1000,1050,1100,1150)	(1050,1100,1150,1200)	(1100,1150,1200,1250)
LV 3 positive capability	(1350,1400,1450,1500)	(1400,1450,1500,1550)	(1450,1500,1550,1600)
LV 1 reverse capability	(550,600,650,700)	(600,650,700,750)	(650,700,750,800)
LV 2 reverse capability	(600,650,700,750)	(650,700,750,800)	(700,750,800,850)
LV 3 reverse capability	(650,700,750,800)	(700,750,800,850)	(750,800,850,900)
LV 3 carbon emission	2300000,2315000,2330000	2315000,2330000,2345000	2330000,2345000,2360000

**Table 7:** Disposal centre-related data.

Disposal center	1	2	3
Coordinates	(102,190)	(199,61)	(283,135)
LV 3 fixed cost (ten thousand yuan)	25,30,35	30,35,40	35,40,45
LV 1 positive capability	(350,400,450,500)	(400,450,500,550)	(450,500,550,600)
LV 2 positive capability	(400,450,500,550)	(450,500,550,600)	(500,550,600,650)
LV 3 positive capability	(450,500,550,600)	(500,550,600,650)	(550,600,650,700)
LV 3 carbon emission	820000,822500, 825000	822500,825000,827500	825000,827500,830000

**Table 8:** Freight-related data.

Freight from production/recovery centre to distribution centre	(0.2,0.3,0.5,0.6)	Freight from the distribution centre to customer area	(0.09,0.10,0.12,0.13)
Freight from customer area to recycling/inspection centre	(0.5,0.6,0.8,0.9)	Freight from recycling/inspection centre to production/recovery centre	(0.3,0.4,0.6,0.7)
Freight from recycling/inspection centre to disposal centre	(0.7,0.8,1.0,1.1)	CO_2_ production per vehicle per kilometer during transportation	(530,540,560,570)
Variable carbon emissions from the operation of the production/recovery centre	(955,965,985,995)	Variable carbon emissions from the operation of the distribution centre	(330,340,360,370)
Variable carbon emissions from the operation of the recycling/inspection centre	(740,750,770,780)	Variable carbon emissions from the operation of the disposal centre	(510,520,540,550)

**Table 9:** Cost savings at joint points with a distribution centre at capacity level 1.

Capacity level of recycling/inspection centre	Joint points		
	1	2	3
1	98750	110000	95000
2	108750	120000	105000
3	118750	130000	115000

### 4.2. Sensitivity analysis and managerial insights

To study the results of the forward/reverse integrated logistics network, let $\hat{r_{k}}$= 0.04 and $\hat{s}$= 0.01. To test the stability of the robust fuzzy model, three cases are discussed that have different levels of conservatism: (1) the certain case: ${\text{Γ}}_{k}^{1}$= 0, ${\text{Γ}}_{l}^{2}$=${\text{Γ}}_{l}^{3}$=0; (2) the intermediate case: ${\text{Γ}}_{k}^{1}$ =  2.5, ${\text{Γ}}_{l}^{2}$ =  ${\text{Γ}}_{l}^{3}$ =  5; and (3) the most conservative case: ${\text{Γ}}_{k}^{1}$= 5, ${\text{Γ}}_{l}^{2}$=${\text{Γ}}_{l}^{3}$=10. First, the robust fuzzy model is solved by using CPLEX12.5.1 with Equations (30) and (31) as targets in the three cases. Tables 12–14 present the analysis results.

**Table 10:** Cost savings at joint points with a distribution centre at capacity level 2.

Capacity level of recycling/inspection centre	Joint points		
	1	2	3
1	108750	120000	105000
2	118750	130000	115000
3	128750	140000	125000

**Table 11:** Cost savings at joint points with a distribution centre at capacity level 3.

Capacity level of recycling/inspection centre	Joint points		
	1	2	3
1	118750	130000	115000
2	128750	140000	125000
3	138750	150000	135000

**Table 12:** Game table of the certain case.

	Objective 1	Objective2
Optimal solution for Objective 1	1633560.60	41871109.37
Optimal solution for Objective 2	2680817.77	27628911.66

**Table 13:** Game table of the intermediate case.

	Objective 1	Objective 2
Optimal solution for Objective 1	1735252.67	42144803.70
Optimal solution for Objective 2	2784185.08	28221095.97

**Table 14:** Game table of the most conservative case.

	Objective 1	Objective2
Optimal solution for Objective 1	1735405.87	42217091.78
Optimal solution for Objective 2	2784335.10	28229419.71

Based on the results in Tables 12–14, objective function ranges are obtained for the three cases and are presented in Table 15.

**Table 15:** Range of objectives.

Objectives	Lower bound	Upper bound
Objective1 in certain case	1633560.60	2680817.77
Objective2 in certain case	27628911.66	41871109.37
Objective1 in intermediate case	1735252.67	2784185.08
Objective2 in intermediate case 2	28221095.97	42144803.70
Objective1 in the most conservative case	1735405.87	2784335.10
Objective2 in the most conservative case	28229419.71	42217091.78

**Fig 2:**
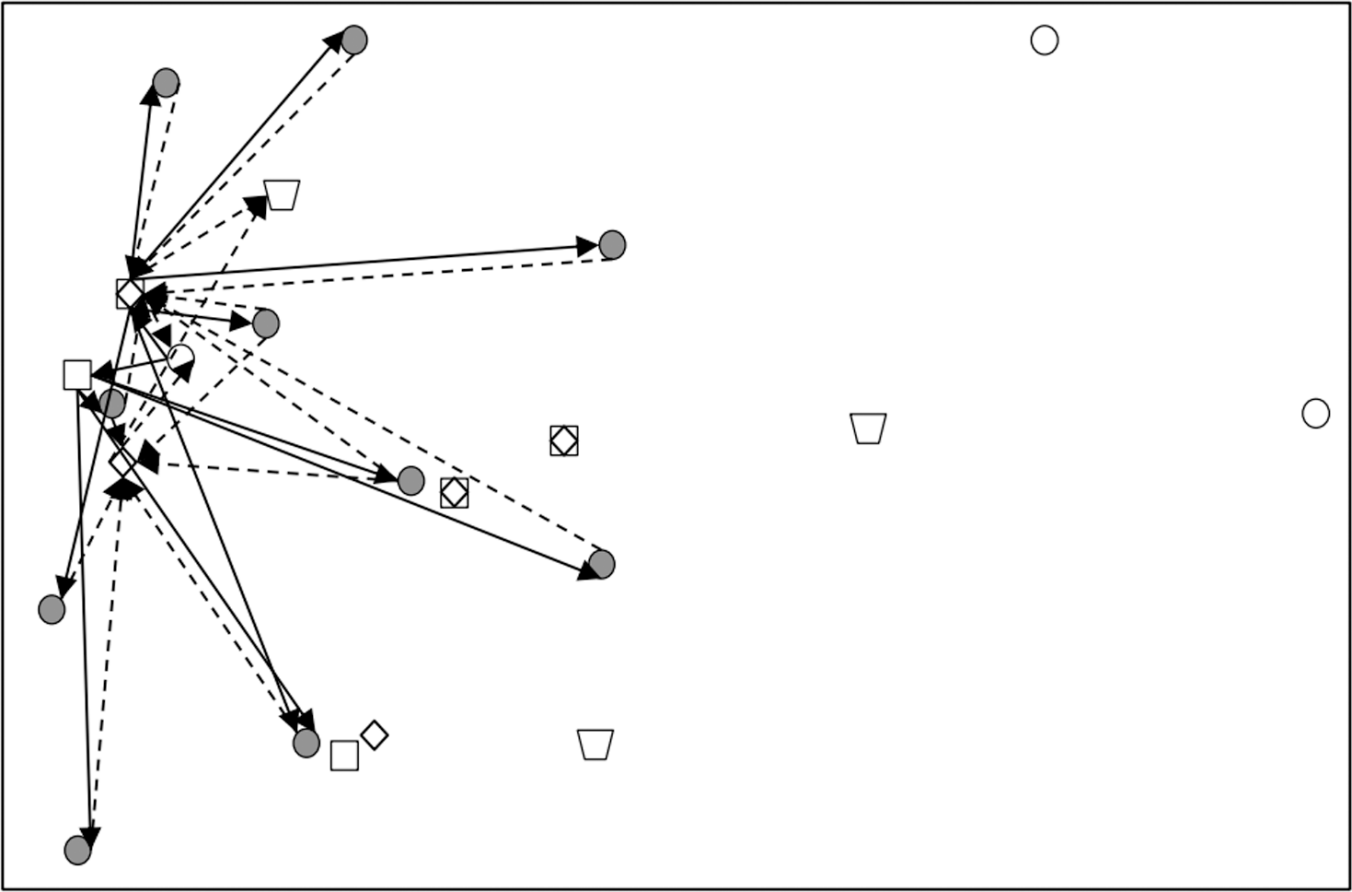
Forward/reverse integrated logistics network diagram of the certain case.

**Fig 3:**
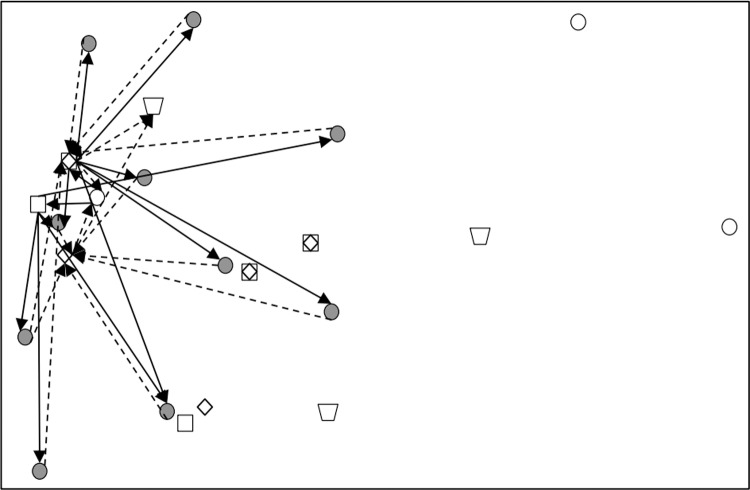
Forward/reverse integrated logistics network diagram of the intermediate case.

**Fig 4:**
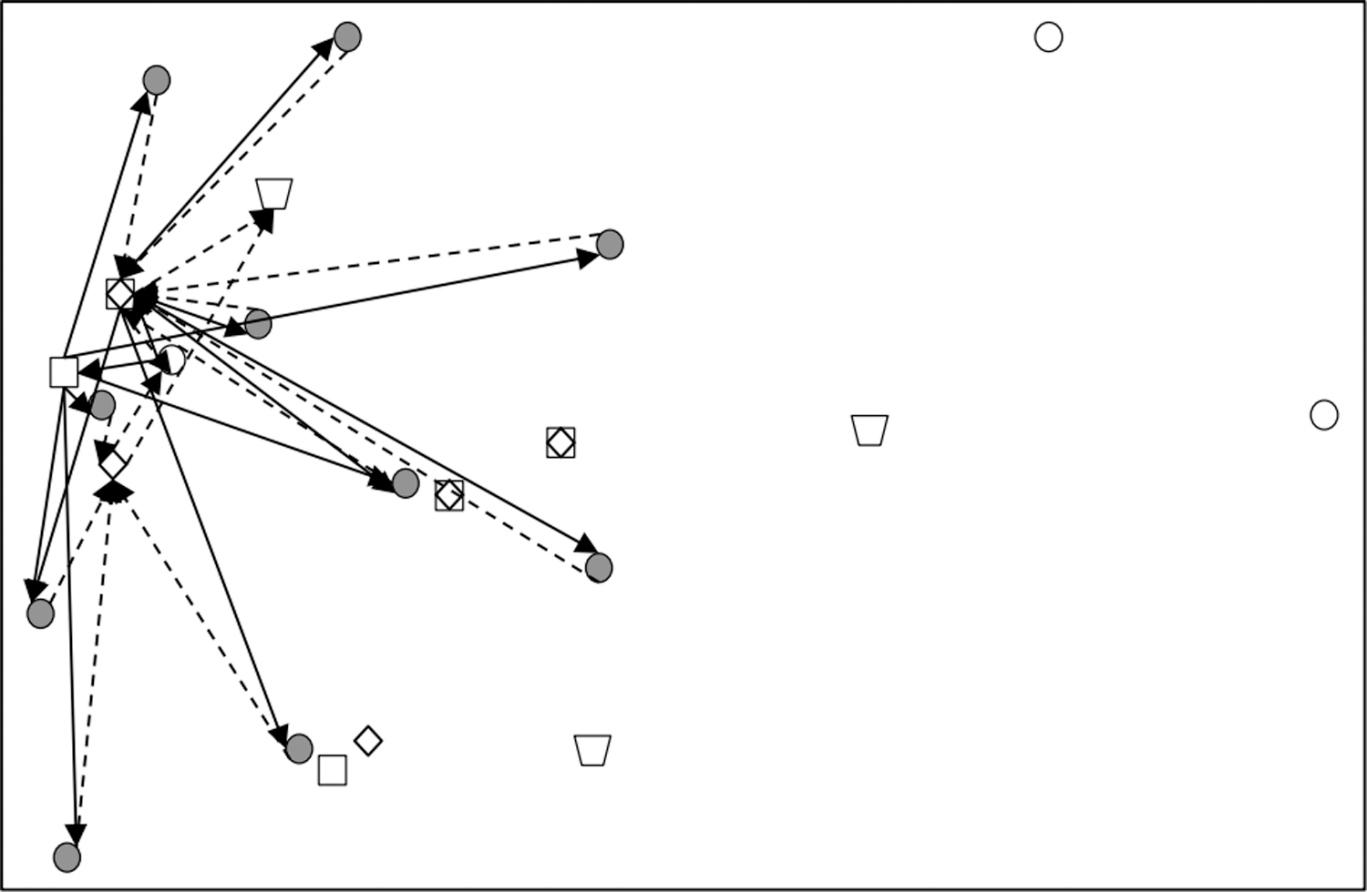
Forward/reverse integrated logistics network diagram of the most conservative case.

Subsequently, CPLEX 12.5.1 was used to solve the established robust fuzzy programming model and the results are presented in Tables 16–18. In Table 17, 00 indicates that this a place is not selected, and 1, 2, 3 represents the level of at which a place choosing this place and the centre is selected). The results are used to construct the graphs and line segments in Figs 2–4, which have the same meaning and are made according to the data (the meanings represented by graphs and line segments in the figure are the same as those in Fig 1). In all three cases, the level of constraint satisfaction $\alpha_{m}$ in all three cases is set to 0.5.

**Table 16:** Optimisation results of the model.

	Target value	$w_{1}^{*}$(Value of Objective1)	$w_{2}^{*}$(Value of Objective2)
Certain case	0.13	1769715.25	29480588.66
Intermediate case	0.14	1876869.77	30100925.73
Most conservative case	0.14	1882260.11	30187775.84

**Table 17:** Site selection for each centre.

	$W_{1}^{n}$	$Y_{1}^{n}$	$Y_{2}^{n}$	$Z_{1}^{n}$	$Z_{2}^{n}$	$V_{1}^{n}$
Certain case	2	1	1	1	2	1
Intermediate case	3	1	2	2	1	1
Most conservative case	3	1	1	1	1	1

Within the fuzzy parameters defined for this scenario, the level of constraint satisfaction is set to 0.5, signifying that there is a 50% likelihood that the aforementioned three cases will satisfy the constraint requirements. This setting reflects the fact that corporate executives are keen to enhance the precision of decision-making processes, which is equivalent to increasing the degree of constraint adherence.

In practical cases, numerous parameters are inherently uncertain, meaning it is unfeasible to attempt to meet all established constraints. Therefore, when parameters are fuzzy numbers, a satisfaction level of 0.5 is realistic. Consequently, decision-making has inherent risks, particularly within an ambiguous context, i.e., one in which it is challenging for corporate executives to assess the validity of a strategy. The model’s incorporation of fuzzy programming techniques adeptly tackles this challenge. Therefore, in this scenario, the conservatism level of the manager’s control to meet the constraint conditions is more than 50%, that is, the manager can judge that the decision obtained in the three cases is the optimal result with a probability of 50%.

In Table 16, the target value fluctuates in response to variations in ${\text{Γ}}_{k}^{1},{\text{Γ}}_{l}^{2}$, and ${\text{Γ}}_{l}^{3}$. The increase in the squared values of ${\text{Γ}}_{l}^{2}$ and ${\text{Γ}}_{l}^{3}$ is attributable to the proliferation of disturbances in the recovery and average processing rates, and to the expansion of the uncertain sets they encompass, which increases amplifying the target value.

Table 17 shows that the quantity of central facility selections remains consistent across the three cases, with each selecting a level 1 distribution centre and a level 1 disposal centre. However, the types of other central facilities selected vary among the cases. The intermediate case selects a level 3 production/recovery centre 1, a level 2 distribution centre 2, a level 2 recycling/inspection centre 1, and level 1 recycling/‘inspection centre 2. In contrast, the definite case selects a level 2 production/recovery centre 1, a level 1 distribution centre 2, a level 1 recycling/inspection centre 1, and a level 2 recycling/inspection centre 2. The most conservative case and the certain case both select a level 1 distribution centre 2 and a level 1 recycling inspection centre 1. However, the most conservative case selects a level 3 production/recovery centre 1 and a level 1 recycling/inspection centre 2, while the certain case selects a level 2 production/recovery centre 1 and a level 2 recycling inspection centre 2. The intermediate case and the most conservative case both select a level 3 production/recovery centre 1 and a level 1 recycling/inspection centre 2. However, the most conservative case selects a level 1 distribution centre 2 and a level 1 recycling/inspection centre 1, while the intermediate case selects a level 2 distribution centre 2 and a level 2 recycling/inspection centre 1.

In Table 18, joint point 2 between the recycling/inspection centre and the distribution centre is selected for the certain and intermediate cases, while no joint point is selected for the most conservative case.

**Table 18:** Selection of joint points.

Joint point	2	4	5
Certain case	1	0	0
Intermediate case	1	0	0
Most conservative case	0	0	0

Finally, Figs 2–4 show that the logistics networks in the three cases have many identical routes. However, they also have a considerable number of different routes. The routes in Figs 2 and 4 are most concentrated on the nodes of recycling/inspection centre 2, while the routes in Fig 3 are most concentrated on the nodes of recycling/inspection centres 1 and 2. These results demonstrate that external disturbances have a significant impact on the flow network structure.

**Fig 5:**
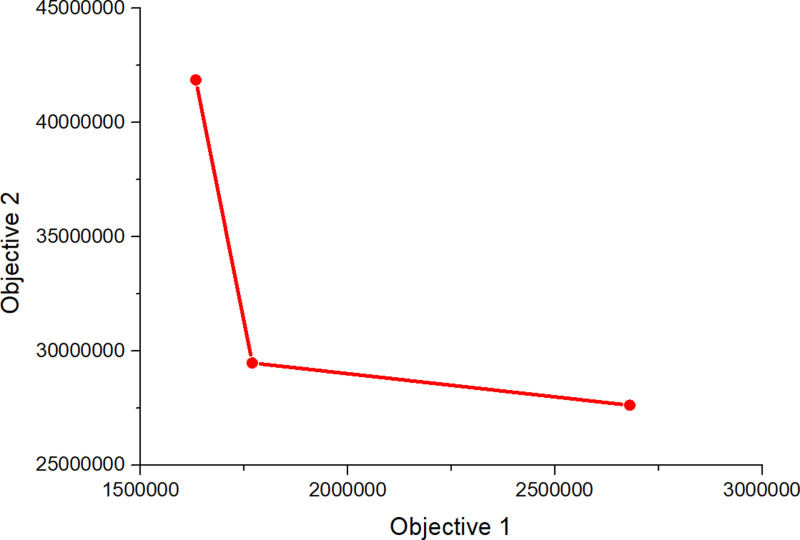
Change curves of two target values in the certain case.

**Fig 6:**
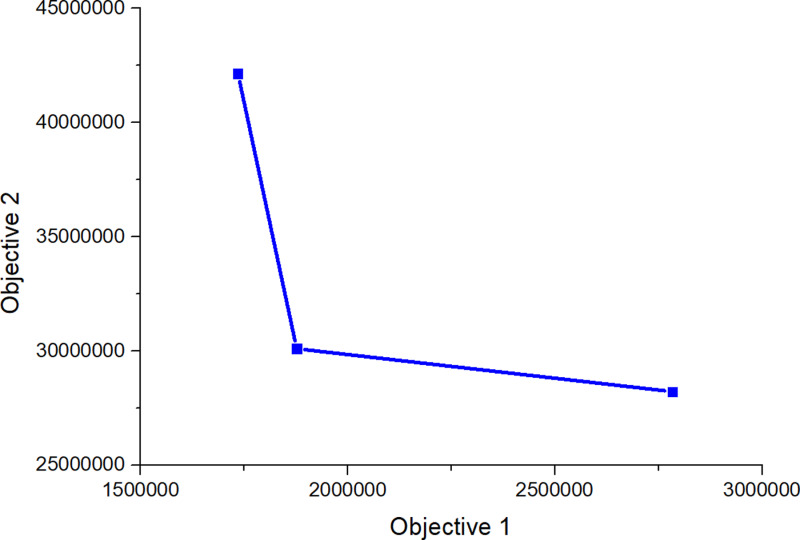
Change curves of two target values in the intermediate case.

**Fig 7:**
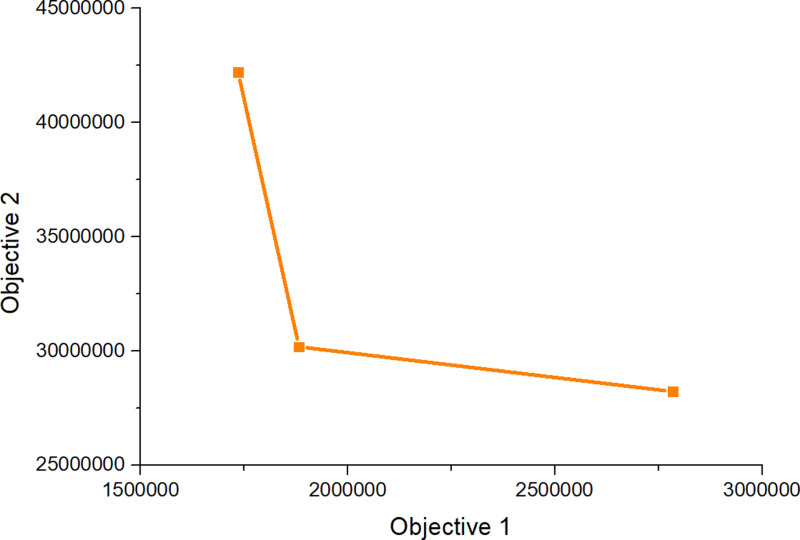
Change curves of two target values in the most conservative case.

By setting the value of δ to 0 ~ 1 (with a scale of 0.1), the optimal values of the two objective functions are obtained in the deterministic, intermediate, and most conservative cases, as shown in Table 19 ~ 21. According to Tables 19–21, generate Figs 5–7 respectively. From Figs 5–7, it can be seen that in all three cases, when the value of δ is 0.5, both objectives can reach fairly low values, indicating that the current two target values belong to satisfactory values that satisfy both target requirements simultaneously.

**Table 19:** Two target values in the Certain case.

δ	Objective1	Objective2
0	2680817.77	27628911.66
0.1	1769787.07	29480456.87
0.2	1769704.91	29480521.84
0.3	1769706.64	29480582.06
0.4	1769704.55	29480589.63
0.5	1769715.25	29480588.66
0.6	1769708.08	29480633.76
0.7	1769707.39	29480535.84
0.8	1769707.77	29480553.48
0.9	1769706.25	29480520.57
1	1633560.60	41871109.37

**Table 20:** Two target values in the intermediate case.

δ	Objective1	Objective2
0	2784185.08	28221095.97
0.1	1876866.42	30100855.28
0.2	1876859.05	30100849.83
0.3	1876884.77	30100816.06
0.4	1876870.98	30100991.08
0.5	1876869.77	30100925.74
0.6	1876864.44	30100874.50
0.7	1876867.66	30100937.18
0.8	1876861.18	30100819.07
0.9	1876863.42	30101019.75
1	1735252.67	42144803.70

**Table 21:** Two target values in the most conservative case.

δ	Objective1	Objective2
0	2784335.10	28229419.71
0.1	1882256.74	30187809.31
0.2	1882256.90	30187874.98
0.3	1882265.19	30187808.66
0.4	1882265.54	30187841.18
0.5	1882260.11	30187775.84
0.6	1882256.32	30187940.39
0.7	1882257.15	30188085.98
0.8	1882256.64	30188502.49
0.9	1882263.65	30188416.95
1	1735405.87	42217091.78

In conclusion, the inherent randomness and fuzziness of parameters influence the positioning of facilities, their interconnecting routes, and the volumes of traffic they accommodate. Therefore, enterprise decision-makers should fully consider their conditions and look forward to future development, grasp the trend of the market, and make appropriate strategic decisions. Hence, the model proposed in this paper has certain applicability, as the obtained solution has superiority and provides data support for enterprise decision-making.

## 5. Concluding remarks

This paper takes the forward/reverse integrated logistics network as its research object and considers green environmental protection. Thus, it uses a robust optimisation and fuzzy programming method and integrates logistics cost and emissions targets to establish a mathematical model. It also converts the model into a deterministic framework, which allows the outcomes of the model’s resolution to substantiate its logical soundness and offer a foundation for corporate strategic decision-making.

The results of this paper indicate that, given the uncertainty parameters, the optimal objective value increases as the disturbance coefficient increases. In addition, when the disturbance coefficient is constant, the optimal objective value increases as the uncertainty parameters increase. When the system’s uncertainty parameters are at their maxima, the system’s objective value is at its maximum, and the system’s robustness is at its minimum. Under different disturbance coefficients, the optimal objective value increases as the uncertainty parameters increase, and the cost difference also increases. Moreover, under different disturbance coefficients, the logistics network continuously changes. Thus, decision-makers can balance the relationship between robustness and optimality by implementing reasonable site selection and route planning.

Unlike previous studies on forward/reverse integrated logistics networks in a single objective determination environment, this paper considers the multi-objective problem and an environment with certainty, randomness, and fuzziness based on a forward/reverse integrated logistics network. Thus, the model devised in this paper is close to reality. Future work can explore reliability and sustainability to ensure the reliable and sustainable development of such logistics networks.
